# Innovative Bio‐based Hydrogel Microspheres Micro‐Cage for Neutrophil Extracellular Traps Scavenging in Diabetic Wound Healing

**DOI:** 10.1002/advs.202401195

**Published:** 2024-04-06

**Authors:** Yongqiang Xiao, Tao Ding, He Fang, Jiawei Lin, Lili Chen, Duan Ma, Tianyu Zhang, Wenguo Cui, Jing Ma

**Affiliations:** ^1^ ENT Institute Department of Facial Plastic and Reconstructive Surgery Eye & ENT Hospital Fudan University Shanghai 200031 P. R. China; ^2^ Department of Orthopaedics Shanghai Key Laboratory for Prevention and Treatment of Bone and Joint Diseases Shanghai Institute of Traumatology and Orthopaedics Ruijin Hospital Shanghai Jiao Tong University School of Medicine 197 Ruijin 2nd Road Shanghai 200025 P. R. China; ^3^ Department of Burn Surgery The First Affiliated Hospital Naval Medical University Shanghai 200433 P. R. China; ^4^ Key Laboratory of Metabolism and Molecular Medicine Ministry of Education Department of Biochemistry and Molecular Biology School of Basic Medical Sciences Fudan University Shanghai P. R. China

**Keywords:** diabetic wound, hydrogel microspheres, inflammation modulation, mesoporous polydopamine, neutrophil extracellular traps

## Abstract

Neutrophil extracellular traps (NETs) seriously impede diabetic wound healing. The disruption or scavenging of NETs using deoxyribonuclease (DNase) or cationic nanoparticles has been limited by liberating trapped bacteria, short half‐life, or potential cytotoxicity. In this study, a positive correlation between the NETs level in diabetic wound exudation and the severity of wound inflammation in diabetic patients is established. Novel NETs scavenging bio‐based hydrogel microspheres ‘micro‐cage’, termed mPDA‐PEI@GelMA, is engineered by integrating methylacrylyl gelatin (GelMA) hydrogel microspheres with cationic polyethyleneimine (PEI)‐functionalized mesoporous polydopamine (mPDA). This unique ‘micro‐cage’ construct is designed to non‐contact scavenge of NETs between nanoparticles and the diabetic wound surface, minimizing biological toxicity and ensuring high biosafety. NETs are introduced into ‘micro‐cage’ along with wound exudation, and cationic mPDA‐PEI immobilizes them inside the ‘micro‐cage’ through a strong binding affinity to the cfDNA web structure. The findings demonstrate that mPDA‐PEI@GelMA effectively mitigates pro‐inflammatory responses associated with diabetic wounds by scavenging NETs both in vivo and in vitro. This work introduces a novel nanoparticle non‐contact NETs scavenging strategy to enhance diabetic wound healing processes, with potential benefits in clinical applications.

## Graphical abstract

This study documents the higher NETs levels in the wound exudation of diabetic patients and their correlation with the the severity of wound inflammation in diabetic patients. A novel NETs scavenging bio‐based hydrogel microsphere ‘micro‐cage’, termed mPDA‐PEI@GelMA, is developed and effectively mitigated pro‐inflammatory responses associated with diabetic wounds by scavenging NETs both in vivo and in vitro.

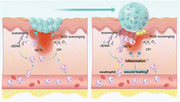

## 1 Introduction

Chronic non‐healing wounds, a critical complication of diabetes, afflict an estimated 19–34% of diabetic patients, impacting ≈83–148 million individuals worldwide.^[^ ^1^ ^]^ These ulcers not only severely disrupt the daily lives of patients but also escalate the risk of amputation and increased mortality. Regrettably, current clinical treatment methods exhibit weaknesses in efficacy and functionality, falling short of achieving the desired therapeutic outcomes. This underscores the urgent need for effective prevention and treatment strategies.^[^ ^2^ ^]^ In diabetic wounds, numerous studies have demonstrated that the healing process is notably impaired due to a prolonged and uncontrolled inflammatory phase,^[^ ^3^ ^]^ triggered by various pathological conditions, which stands as a key hallmark of impaired diabetic wounds healing.^[^ ^4^ ^]^

Recent research has elucidated that a central factor in the pathogenesis of dysregulated inflammation in diabetic wounds healing is the excessive release of neutrophil extracellular traps (NETs) from neutrophils, significantly contributing to the delayed healing of diabetic wounds. NETs form an extracellular fibers network comprising a cell‐free DNA (cfDNA) backbone with a diameter of 15–17nm and spherical structural domains of ≈25nm.^[^ ^5^ ^]^ Properly regulated NETs play a constructive role in wound healing by capturing and neutralizing pathogenic microorganisms and inhibiting infection‐related inflammation. However, in a hyperglycemic environment of diabetic wounds, prolonged excessive activation and dysregulated apoptosis of neutrophils lead to enhanced susceptibility to NETosis, and formation progress of NETs, resulting in higher NETs release compared to healthy controls. NETs may aggravate inflammation and cause persistent injury to wound tissues^[^ ^6^ ^]^ by promoting endothelial‐to‐mesenchymal transition in a Hippo‐dependent pathway, leading to reduced angiogenesis,^[^ ^7^ ^]^ up‐regulating the inflammatory response through the cfDNA web or histone‐induced toll‐like receptor 9 (TLR‐9) / nuclear factor kappa‐B signaling pathway activation.^[^ ^8^ ^]^

Despite the detrimental impact of NETs on diabetic wounds healing, therapeutic strategies for their effective scavenging or inhibition remain limited in clinical settings. Previous research has explored the disruption of NETs using deoxyribonuclease (DNase) in experimental models of diabetic wounds treatment—an approach approved by the US Food and Drug Administration (FDA) for treating inflammation‐related diseases.^[^ ^6a^ ^]^ However, its clinical application has been hindered by significant side effects, such as the liberation of bacteria trapped within NETs.^[^ ^9^ ^]^ Considering that cfDNA webs are the dominant structure of NETs, the use of cationic nanoparticles for scavenging NETs by targeting cfDNA web through strong electrostatic interaction has gained prominence as a viable approach to attenuate the dysregulated inflammatory response in diabetic wounds. This cfDNA scavenging strategy, already explored in various inflammation‐related diseases,^[^ ^10^ ^]^ suggests that may assist in the scavenging of NETs by binding NET‐cfDNA, thereby reducing the harmful effects of NETs in diabetic wounds healing.

Compared to using DNase, this NETs‐DNA scavenging strategy offers certain advantages. By selectively targeting and binding the cfDNA components of NETs, without fully degrading the DNA backbone, the risk of releasing trapped bacteria and promoting inflammation may be reduced. However, nanoparticles’ small size and unique physicochemical properties can make them highly reactive and capable of penetrating biological barriers into systemic circulation. This means that nanoparticles applied to diabetic wounds could potentially spread throughout the body and interact with other tissues, which could have unintended consequences and lead to unpredictable clinical outcomes.^[^ ^11^ ^]^

**Figure 1:**
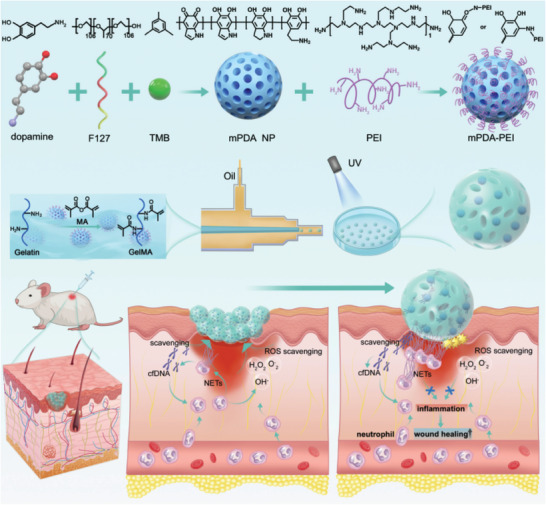
Synthesis process of neutrophil extracellular traps (NETs) scavenger ‘micro‐cage’ mPDA‐PEI@GelMA and its application in treating wounds in diabetic mice through NETs scavenging strategy. NETs were introduced into the ‘micro‐cage’ along with wound exudation, and the cationic mPDA‐PEI immobilizes them inside the ‘micro‐cage’ through a robust binding affinity to the cfDNA web structure.

Therefore, our study is pioneering in utilizing clinical patient samples to establish a positive correlation between local NETs‐specific marker levels in diabetic wounds exudation and the severity of diabetic wounds. This correlation further strengthens the case for considering NETs as a potential therapeutic target for diabetic wounds. Recognizing the challenges associated with the direct application of nanoparticles on the wound surface, we have developed a non‐contact NETs scavenging strategy employing a specialized hydrogel microsphere known as the ‘micro cage’ scavenger, named mPDA‐PEI@GelMA. This strategy involves the integration of methylacrylyl gelatin (GelMA) hydrogel microspheres with cationic polyethyleneimine (PEI)‐functionalized mesoporous polydopamine (mPDA). GelMA microspheres, known for their robust swelling properties,^[^ ^12^ ^]^ actively and efficiently absorb wound exudation containing substantial amounts of NETs. The cationic mPDA‐PEI, serving as the core components of ‘micro cage’, loaded inside the microspheres, firmly captures NETs through a strong binding affinity to the cfDNA web structure. This approach achieves non‐contact NETs scavenging between nanomaterials and the wound surface, minimizing biological toxicity and ensuring high biosafety. Importantly, the micron size of mPDA‐PEI's allows for extended residency and treatment time in the wound.^[^ ^11a^ ^]^ Meanwhile, the injectable characteristics of GelMA microspheres make them particularly conducive to addressing complex diabetic deep and sinus wounds, rendering them an ideal biological material for diabetic wounds.^[^ ^12^, ^13^ ^]^ Our investigation delves into the binding affinity of mPDA‐PEI@GelMA to NETs‐cfDNA and its effectiveness in reducing pro‐inflammatory neutrophil phenotypes and NETs generation. The study culminates in assessing the therapeutic efficacy of this approach in a diabetic wounds murine model, supporting the hypothesis that a nanoparticulate GelMA microsphere, integrated as a NETs scavenger ‘micro cage’, could significantly accelerate diabetic wounds healing. (**Figure** **1**).

## 2 Results and Discussion

### 2.1 The Levels of Neutrophil Extracellular Traps in Diabetic Wound Exudation Intricately Correlate With the Severity of Diabetic Wound Injuries

Diabetic wounds, characterized by slow healing and susceptibility to infection, pose a risk of complications, such as amputation and increased mortality. NETs are released by neutrophils to trap and kill pathogens. However, dysregulated NETs formation can contribute to tissue damage and impaired wound healing.^[^ ^6^ ^]^ Studies have demonstrated that citrullinated histone 3 DNA (CitH3‐DNA) complex can serve as a marker of NETs.^[^ ^14^ ^]^ When NETs are formed, they also release cfDNA outside the neutrophiles, which can be detected and measured as an indicator of NETs activity. Skin tissue injury could also lead to cellular necrosis and compromised membrane integrity, triggering the release of cfDNA. This plays a pivotal role in immunomodulation and incites inflammatory responses, hindering the healing process in diabetic wounds.^[^ ^15^ ^]^ To elucidate the dynamics of CitH3‐DNA complexes and cfDNA in the wound exudation of patients with burns and diabetes, a cohort study was conducted involving the collection of 48 samples from the Department of Burn Surgery at the Changhai Hospital of Naval Medical University. These samples included 33 exudation samples from 21 patients with burn wounds and 12 patients with diabetic wounds (Figure S1, Supporting Information), along with 15 plasma samples from healthy controls. Quantitative analyses of CitH3‐DNA complexes, cfDNA, myeloperoxidase (MPO), cell‐free microRNA (cfmiRNA), and a spectrum of inflammatory cytokines, including Interleukin‐6 (IL‐6), Tumor Necrosis Factor‐α (TNF‐α), Interleukin‐1 beta (IL‐1β), and Interferon‐gamma (IFN‐γ), were performed using specific assay kits.

**Figure 2:**
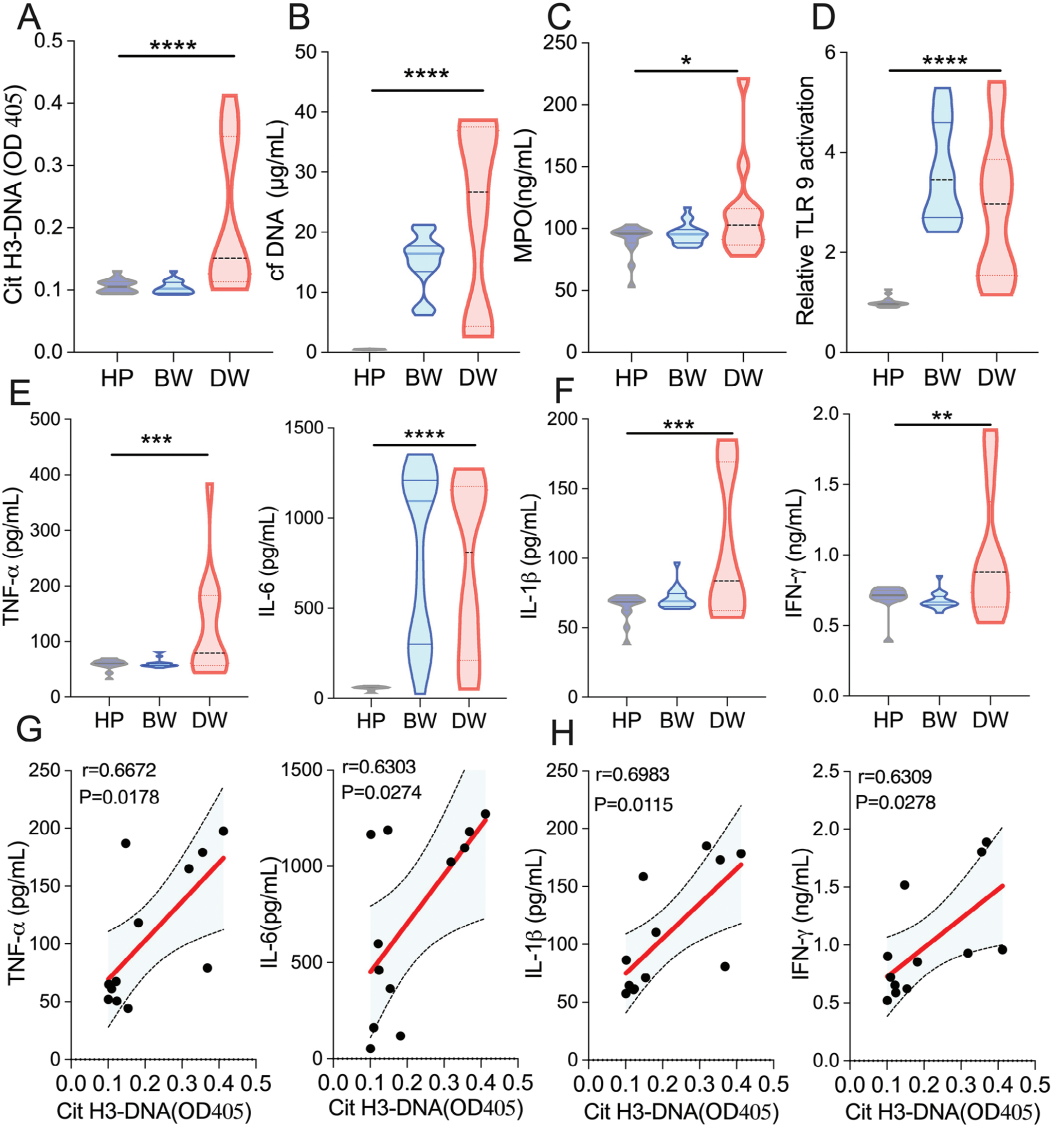
The relationship between NETs and features of burn and diabetic wound patients. (A) The citrullinated histone‐3‐DNA (CitH3‐DNA) complex level; (B) The cfDNA concentrations and (C) The MPO concentrations, in plasma from healthy volunteers (HP, *n =* 15) and wound exudation from patients with burn wounds (BW, *n =* 21) and diabetic wounds (DW, *n =* 12). (D) Activation of TLR 9 reporter cells by plasma from healthy volunteers (*n =* 15) and wound exudation from patients with burn (*n =* 21) and diabetic wound (*n =* 12). Data represents the mean ± S.D. (One‐way ANOVA, *p<0.05, **p<0.01, ***p<0.001, ****p<0.0001). (E and F) The levels of TNF‐α, IL‐6, IL‐1β, and IFN‐γ in plasma from healthy volunteers (*n =* 15) and wound exudation from patients with burn (*n =* 21) and diabetic (*n =* 12). Data represents the mean ± S.D. (One‐way ANOVA, *p<0.05, **p<0.01, ***p<0.001, ****p<0.0001). (G and H) Scatter plot of CitH3‐DNA complex and inflammatory cytokines levels of diabetic wound (*n =* 12). The red line is the fitted regression line, and the light blue shading around it is the 95% confidence interval. Coefficient ‘r’ represents Spearman's correlation between CitH3‐DNA complex levels and TNF‐α, IL‐6, IL‐1β, and IFN‐γ.

The findings revealed a marked elevation of CitH3‐DNA complexes, cfDNA, and cfmiRNA levels in wound exudation from both burn and diabetic patients compared to healthy plasma controls (cfDNA: 15.07 ± 4.65 and 23.11 ± 15.34 µg mL^−1^ versus 0.49 ± 0.06 µg mL^−1^; cfmiRNA: 33.38 ± 18.18 and 52.91 ± 13.07 µg mL^−1^ versus 1.32 ± 0.24 µg mL^−1^) (**Figure** **2**A,B; Figure S2A, Supporting Information). Importantly, these levels surpassed those reported in plasma from patients with sepsis, trauma, and periodontitis.^[^ ^10^, ^16^ ^]^ Notably, CitH3‐DNA complexes, cfDNA, and cfmiRNA concentrations were significantly higher in diabetic wound exudation compared to burn wound exudation, suggesting sustained tissue damage in diabetic wounds. MPO, another specific marker of NETs, also showed a significant increase in diabetic wounds exudation (Figure 2C Supporting Information), together with elevated CitH3‐DNA complexes and cfDNA suggesting increased NETs in diabetic wounds exudation.

Moreover, wound exudation from both patient groups induced more robust activation of TLR 3, 8, and 9 compared to plasma from healthy controls (Figure 2D; Figure S2B,C, Supporting Information), suggesting a role in triggering immune cascades and uncontrolled inflammation.^[^ ^17^ ^]^ Furthermore, the levels of inflammatory cytokines (TNF‐α, IL‐1β, and IFN‐γ) in diabetic wounds exudation were substantially higher than those in burn wound exudation and healthy plasma, indicating a state of chronic hyperinflammation. (Figure 2E,F) IL‐6 levels surged rapidly and significantly in acute burn wound exudation (Figure 2E), identifying it as a critical marker and regulator in acute wound inflammation.^[^ ^18^ ^]^ However, persistently high expression of IL‐6 in chronic wounds indicated a failure to regulate the transition to proliferative or remodeling phase adequately, leading to chronic inflammation and impaired wound healing.^[^ ^19^ ^]^

Further analysis examined the correlation between CitH3‐DNA complexes and cfDNA levels and inflammatory cytokines (IL‐6, TNF‐α, IL‐1β, and IFN‐γ) in wounds exudation (Figure 2G,H; Figures S3A–D and S4A–D, Supporting Information). A strong positive correlation was observed between CitH3‐DNA complexes and these inflammatory cytokines in diabetic wounds exudation (Figure 2G,H). A strong positive correlation was also observed between cfDNA and diabetic or total wound exudation (Figures S3 and S4, Supporting Information), suggesting that elevated local cfDNA levels may contribute to the chronic inflammatory cascade in diabetic wounds healing. All these data highlight the potential role of NETs in the pathophysiology of diabetic wounds and in developing targeted therapies to promote better outcomes.

### 2.2 Synthesis and Characterization of mPDA‐PEI@GelMA

**Figure 3:**
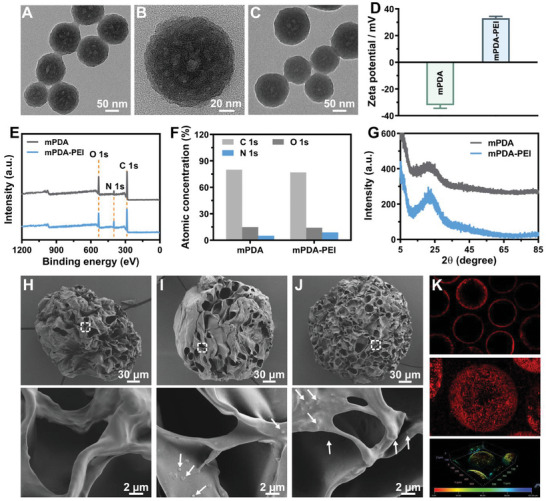
Synthesis and characterization of mPDA‐PEI@GelMA. (A–C) TEM images of mPDA, including a close‐up view of a selected mPDA, and mPDA‐PEI NPs. (D) The zeta potentials of mPDA and mPDA‐PEI. (E,F) XPS survey of as well as the atomic concentration of C 1s, O 1s, and N 1s. (G) XRD patterns of mPDA and mPDA‐PEI NPs. (H–J) SEM images of GelMA, mPDA@GelMA, and mPDA‐PEI@GelMA microspheres, along with the enlarged images of the selected area (white rectangle). The white arrow indicates the presence of mPDA or mPDA‐PEI nanoparticles within the GelMA hydrogel microspheres. (K) CLSM image, overlay of the Z‐stack scanning image, and the 3D image of Cy5‐PEG labeled mPDA‐PEI@GelMA.

Given the established correlation between NETs and inflammatory cytokine production in diabetic wounds, along with the involvement of TLR9 activation in chronic inflammatory responses,^[^ ^7^, ^8^ ^]^ we first designed an effective nanoparticulate NETs scavenger named ‘mPDA‐PEI@GelMA’ for binding the NETs‐cfDNA structure. The selection of mPDA as the core material for mPDA‐PEI@GelMA was based on its favorable characteristics. Polydopamine nanoparticles (PDA NPs), synthesized through dopamine self‐assembly, are known for their biocompatibility and exceptional photoelectric conversion efficiency, making them suitable for gene and drug delivery. PDA NPs also possess active catechol/quinone, amine, and imine groups, allowing for binding through various interactions, such as π‐π stacking, hydrogen bonding, and electrostatic attraction.^[^ ^20^ ^]^ The mPDA NPs provide an enlarged contact area, combining a vast surface area with metal‐chelating properties and broad‐spectrum photothermal transduction for efficient loading. The synthesis of mPDA and mPDA‐PEI NPs followed established methods,^[^ ^20^, ^21^ ^]^ resulting in synthetic NPs with a regularly ordered mesoporous structure and a size distribution of 107.3 ± 5.03 nm, as confirmed by transmission electron microscopy (TEM) (**Figure** **3**A–C). The charge characteristics were compared between the negatively charged mPDA (‐32.13 ± 2.329) and the positively charged PEI grafted mPDA (mPDA‐PEI) (33.03 ± 1.422) (Figure 3D). X‐ray photoelectronic spectroscopy (XPS) data demonstrated the co‐existence of elements C, N, and O in both mPDA and mPDA‐PEI (Figure 3E). The concentration of N 1s in mPDA‐PEI showed a slight increase compared to mPDA, likely attributed to the higher N ratio in PEI molecules (Figure 3F). X‐ray diffraction (XRD) illustrated a high similarity in phase characteristics between mPDA and mPDA‐PEI (Figure 3G), confirming the structural integrity of PDA after modification with PEI. These comprehensive characterizations collectively confirm the successful PEI coating of the mPDA NPs, laying the foundation for the development of an efficient NETs scavenger for diabetic wounds.

However, a significant challenge facing the clinical application of nanoparticles is their small size and unique physicochemical properties. These features can make them highly reactive and capable of penetrating biological skin barriers into the systemic circulation, potentially leading to unintended consequences and unpredictable clinical outcomes.^[^ ^11^, ^22^ ^]^ In our study, we addressed these challenges by employing GelMA hydrogel microspheres as carriers. This strategy created a ‘micro‐cage’ for mPDA‐PEI in wound applications, aiming to prolong its retention time and mitigate potential cytotoxicity concerns. The GelMA microspheres were fabricated using microfluidic technology (Figure 1A),^[^ ^13^, ^23^ ^]^ yielding white granules with excellent dispersion and sedimentation properties (Figures S9 and S11, Supporting Information). The mPDA‐PEI@GelMA microspheres, with a hydrodynamic diameter of ≈200 µm, were prepared using microfluidic technology. Scanning electron microscopy (SEM) revealed a dense three‐dimensional network structure and the presence of mPDA or mPDA‐PEI nanoparticles within the GelMA hydrogel microspheres. (Figure 3H–J), while bright‐field microscopy further confirmed their biodegradability over time (Figure S9, Supporting Information). Confocal laser scanning microscopy (CLSM) provided visual confirmation of the successful immobilization of mPDA‐PEI within the GelMA microspheres, indicating effective retention of mPDA‐PEI nanoparticles within the GelMA network. These comprehensive findings collectively affirm the successful synthesis of the nanoparticulate NETs‐cfDNA scavenger ‘micro‐cage’, utilizing GelMA microspheres integrated with cationic PEI‐functionalized mPDA. This innovative approach showcases its potential for diabetic wounds treatment applications, addressing concerns related to stability and biocompatibility.

### 2.3 The Efficiency of mPDA‐PEI@GelMA in Scavenging cfDNA

**Figure 4:**
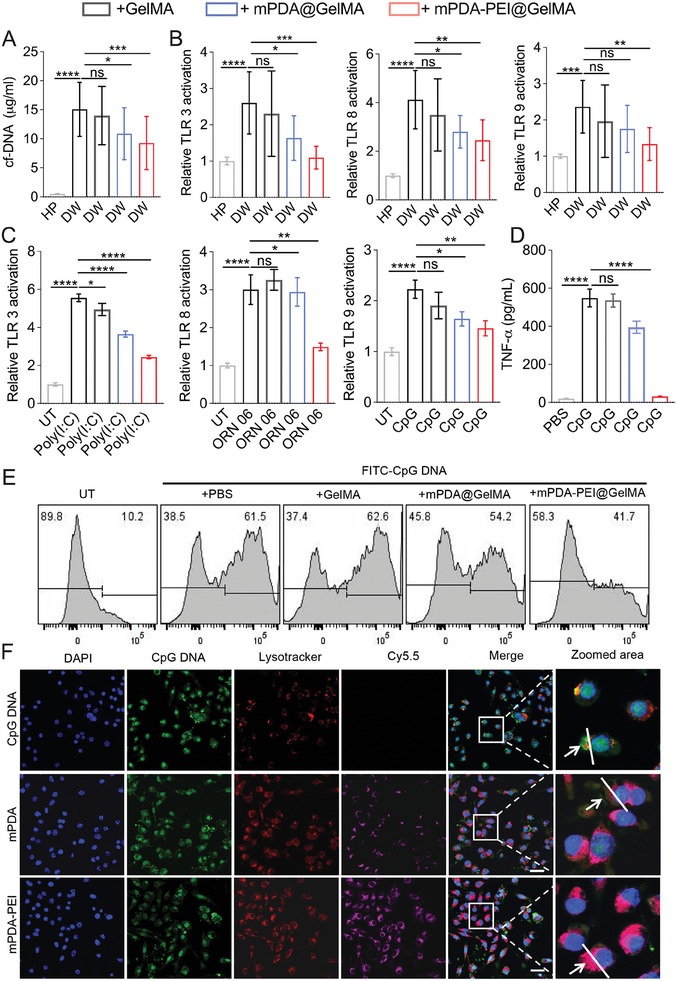
The mPDA‐PEI@GelMA blocks diabetic wound‐related TLR proinflammatory response in vitro. (A) The cfDNA binding efficiency of mPDA‐PEI@GelMA in the diabetic wound exudation. (B) The activation of HEK‐TLR 3, 8, and 9 reporter cells by wound exudation from patients with diabetic wounds. (C) The activation of HEK‐TLR 3, 8, and 9 reporter cells by Poly(I:C), ORN 06, or CpG DNA (1 µg mL^−1^). (D) The TNF‐α generation of Raw 264.7 induced by CpG DNA (1 µg mL^−1^) for 24 h. (E) The endocytosis of FITC‐labeled CpG DNA by macrophage in the absence or presence of GelMA, mPDA@ GelMA, or mPDA‐PEI@ GelMA for 24 h. (F) CLSM images of RAW264.7 cells after 24 h incubation with CpG DNA + mPDA or CpG + mPDA‐PEI. CpG DNA was labeled with FITC; mPDA or mPDA‐PEI were labeled with Cy5‐PEG and lysosomes were labeled with Lysotracker. Scale bars, 10 µm. (Data are presented as means ± SD; *p< 0.05, **p< 0.01, ***p< 0.001, ****p< 0.0001 assessed by one‐way ANOVA with Tukey's multiple comparison test).

Upon the successful fabrication of the NETs scavenger ‘micro‐cage’, mPDA‐PEI@GelMA, we initiated a comprehensive assessment of its cfDNA scavenging capabilities within wound exudation. **Figure** **4**A vividly illustrates the superior cfDNA scavenging performance of mPDA‐PEI@GelMA, surpassing the efficacy of both GelMA and mPDA@GelMA. To further validate its therapeutic potential, we investigated the impact of mPDA‐PEI@GelMA on TLR activation, confirming its immune‐modulatory effects. Utilizing HEK‐TLR3,TLR8, andTLR9 reporter cells, we exposed them to a combination of wound exudation and specific TLR agonists_poly(I:C) dsRNA, ORN06 ssRNA, and CpG DNA. The compelling results depicted in Figure 4B,C unequivocally demonstrate that mPDA‐PEI@GelMA exhibits superior inhibitory effects on the activation of TLR 3, 8, and 9, induced by both wound exudation and their respective nucleic acid ligands, in comparison to GelMA and mPDA@GelMA. Furthermore, the immunomodulatory prowess of mPDA‐PEI@GelMA was evidenced by a substantial reduction in the release of inflammatory cytokines from RAW264.7 cells in response to CpG DNA, as highlighted in Figure 4D.

**Figure 5:**
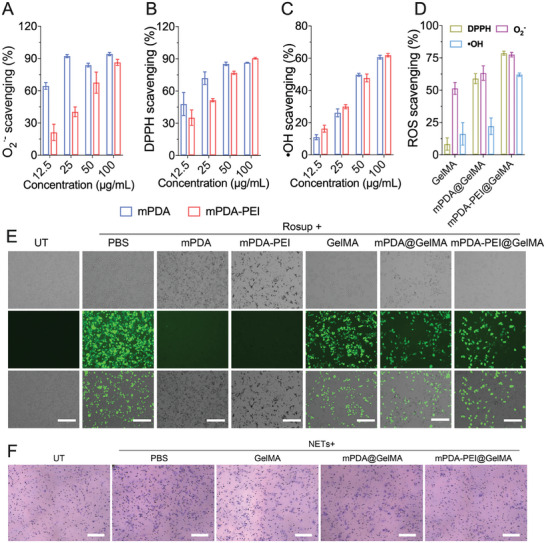
The ROS‐scavenging efficiency by mPDA‐PEI@GelMA. (A–C) ROS scavenging efficiency of mPDA and mPDA‐PEI toward •O^2–^, DPPH and •OH. (D) ROS scavenging efficiency of GelMA, mPDA@GelMA, and mPDA‐PEI@GelMA toward O^2•−^, DPPH, and •OH. (E) Intracellular ROS fluorescence photographs in the Rosup‐treated Raw 264.7 cells treated with mPDA, mPDA‐PEI, GelMA, mPDA@GelMA, and mPDA‐PEI@GelMA pretreated for 24h. Scale bars, 200 µm. (F) Images of RAW264.7 cells migration induced by PMA‐activated neutrophils after 24 h of incubation with mPDA‐PEI@GelMA. Scale bars, 150 µm.

Given the pivotal role of NETs released local cfDNA in activating the TLR9 signaling pathway in chronic diabetic wounds through endocytosis by inflammatory cells, our investigation delved deeper into the potential of mPDA‐PEI@GelMA for cfDNA scavenging in this context. Leveraging the water absorption and expansion properties of GelMA hydrogel microspheres,^[^ ^12^ ^]^ we postulated that these microspheres could facilitate the adsorption of cationic mPDA‐PEI and encapsulate cfDNA, thereby hindering its endocytosis by inflammatory macrophages and subsequently attenuating the inflammatory response. To simulate this scenario, we employed CpG oligodeoxynucleotides (CpG‐OND) as cfDNA analogs to assess both the endocytosis of CpG‐OND by RAW 264.7 macrophage cells and the inhibitory effect of mPDA‐PEI@GelMA. As illustrated in Figure 4E, mPDA‐PEI@GelMA exhibited a significantly superior restriction of CpG DNA endocytosis by RAW 264.7 macrophage cells compared to either mPDA@GelMA or GelMA alone. Furthermore, we delved into the internalization dynamics of Cy5‐PEG‐labeled mPDA‐PEI and FITC‐labeled CpG DNA into RAW 264.7 cells and their capacity to block CpG‐induced TLR9 activation. Notably, Cy5‐PEG‐labeled mPDA and mPDA‐PEI were observed to accumulate in endolysosomes, as visualized through CLSM (**Figure** **5**F). Strikingly, in comparison to mPDA (Cy5‐PEG‐labeled), mPDA‐PEI (Cy5‐PEG‐labeled) exhibited a more pronounced co‐localization of mPDA‐PEI (Cy5‐PEG‐labeled) with FITC‐labeled CpG DNA in the endolysosomal compartments (Figure 5F). This observation underscores the enhanced binding of mPDA‐PEI to CpG DNA, thereby inhibiting the recognition of CpG DNA by TLR9.

### 2.4 The Reactive Oxygen Species Scavenging Efficiency of mPDA‐PEI@GelMA

Excessive oxidative stress, primarily induced by reactive oxygen species (ROS), such as hydrogen peroxide (H_2_O_2_), hydroxyl radicals (•OH), and superoxide anion (•O^2–^), plays a detrimental role in cellular damage in diabetic wounds. This oxidative stress not only compromises skin cell survival but also induces a pro‐inflammatory macrophage phenotype, sustaining a chronic pro‐inflammatory environment and impeding the natural wound healing process.^[^ ^24^ ^]^ Recognizing the significance of addressing oxidative stress in diabetic wound management, ROS scavenging strategies have emerged as promising treatments, with PDA standing out as a noteworthy candidate.^[^ ^25^ ^]^ PDA, rich in reducing groups like phenol and catechol, exhibits excellent redox capacity and serves as an effective ROS scavenger by donating free radical electrons.^[^ ^26^ ^]^ In particular, mPDA NPs, known for their enhanced active site accessibility, have garnered considerable attention in this context.^[^ ^27^ ^]^

We then investigated the ROS scavenging capabilities of various materials (mPDA, mPDA‐PEI, mPDA@GelMA, mPDA‐PEI@GelMA, and GelMA) against •OH, •O^2–^, and DPPH radicals using  corresponding ROS analysis kits. To assess their radical scavenging abilities, we compared the UV−vis absorbance of samples with a control radical solution. Notably, mPDA‐PEI exhibited slightly weaker •O^2–^ and DPPH scavenging compared to mPDA, attributed to active site loss post‐PEI coating. However, it still achieved a scavenging rate of ≈90% in a dose‐dependent manner (Figure 5A,B). The •OH scavenging ability of both mPDA and mPDA‐PEI was similar and displayed a dose‐dependent trend (Figure 5C). Upon integration with GelMA microspheres, the ROS scavenging efficiency of mPDA‐PEI@GelMA toward •OH, •O^2–^, and DPPH slightly decreased due to active site loss but still maintained a free radical scavenging rate of ≈ 70%, surpassing both mPDA@GelMA and pure GelMA (Figure 5D). Despite the reaction‐strict control of reaction conditions strictly controlled, due to the electrostatic interaction between the positively charged mPDA‐PEl and GelMA microspheres may lead to a higher concentration of mPDA‐PEI nanoparticles within GelMA compared to mPDA, which may be larger than that of mPDA negatively charged under neutral conditions. This disparity results, which results in a slightly higher efficiency of ROS removal for the efficiency of mPDA‐PEI@GelMA compared to mPDA@GelMA(Figure 5D). Intracellular ROS scavenging by mPDA and mPDA‐PEI in Rogust‐stimulated RAW 264.7 cells was complete (Figure 5E). However, the efficiency of mPDA@GelMA and mPDA‐PEI@GelMA within these cells was significantly reduced, likely due to the diminished release of mPDA and mPDA‐PEI from the microspheres into the cells. These results collectively demonstrate that the NETs scavenger ‘micro‐cage’ is also effective in ROS scavenging, further highlighting its potential in wound treatment applications.

Furthermore, in early‐phase dermal wounds, neutrophils, serving as primary cells, release chemokines upon activation. This process attracts monocytes/macrophages and contributes to a chronic inflammatory state through excessive pro‐inflammatory cytokine production (e.g., IL‐12, IL‐1β, IL‐6, TNF‐α, iNOS).^[^ ^28^ ^]^ A promising strategy for alleviating chronic inflammation in diabetic wounds involves reducing neutrophil‐induced macrophage recruitment. In our study, we investigated whether mPDA‐PEI@GelMA could inhibit macrophage migration induced by Phorbol myristate acetate (PMA)‐activated neutrophils using transwell assays. The results indicated that PMA‐activated neutrophils recruited numerous macrophages across the transwell chamber due to chemotactic attractants. However, mPDA‐PEI@GelMA significantly reduced this migration (Figure 5F). Consequently, mPDA‐PEI@GelMA not only inhibits nucleic acid‐initiated TLR activation but also mitigates activated neutrophil‐induced macrophage migration.

### 2.5 mPDA‐PEI@GelMA Suppressed Neutrophils Polarization to the N1 Subtype and NETs Generation

Contrary to the traditional perception of neutrophils as metabolically inert due to their brief lifespan, recent evidence has emphasized that neutrophil behavior switches between the pro‐inflammatory and cytotoxic nature of the N1 subtype of neutrophils, and an anti‐inflammatory, pro‐resolution phenotype, termed the N2 subtype.^[^ ^29^ ^]^ The N1 subtype is also known for its heightened NETs formation,^[^ ^5^, ^30^ ^]^ contributing to the delayed healing of diabetic wounds.^[^ ^31^ ^]^ Following the confirmation of mPDA‐PEI@ GelMA's ability to scavenge cfDNA in diabetic wounds exudation, we investigated its impact on neutrophil polarization to the N1 subtype and its subsequent effect on NETs formation.

**Figure 6:**
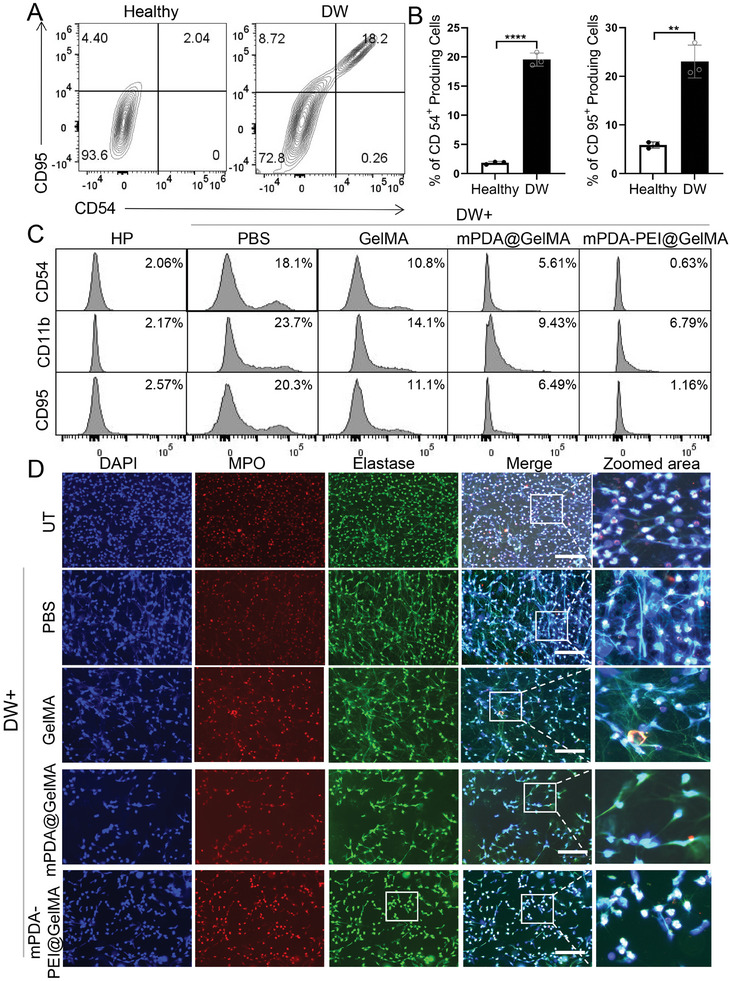
The effects of mPDA‐PEI@GelMA on neutrophil polarization and NETs generation. (A,B) Expression of the N1 markers CD 95 and CD54 in healthy human neutrophil cells activated by diabetic wound exudation or healthy plasma. Data are means ± SD; differences were assessed by two‐tailed Student's t‐test (*n =* 3 samples per group). (C) Effects of diabetic wound exudation on expression of CD95, CD54, and CD11B in healthy human neutrophil cells, along with the treatment effects of mPDA‐PEI@GelMA. (D) Effects of NETs scavenger ‘micro‐cage’ on the diabetic wound exudation‐induced NETs generation. (*p<0.05, **p<0.01, ***p<0.001, ****p<0.0001) DW represents the diabetic wounds.

To mimic the diabetic wounds microenvironment, human neutrophils were exposed to diabetic wounds exudation, and the subsequent effects of mPDA‐PEI@GelMA treatment on neutrophil phenotypic changes were analyzed. The findings, as illustrated in **Figure** **6**A,B, revealed a significant increase in the proportion of CD95+(ICAM‐1+) and CD54+ cells (markers of the N1 neutrophil subtype) in response to diabetic wounds exudation compared to healthy plasma. This indicates a pronounced polarization of neutrophils toward the N1 subtype. However, mPDA‐PEI@GelMA was observed to more effectively reduce the percentage of N1 subtype neutrophils than mPDA@GelMA and GelMA (Figure 6C).

Furthermore, as CD95+ neutrophils are associated with increased ROS and NETs production,^[^ ^5^, ^30^ ^]^ we assessed NETs formation under various conditions, including exposure to diabetic wounds exudation and mPDA‐PEI@GelMA treatment. As depicted in Figure 6D, diabetic wounds exudation markedly increased NETs formation, as indicated by MPO, Elastase, and DAPI labeling, signifying an inclination of N1‐type neutrophils toward NETs generation. Notably, the inclusion of mPDA‐PEI@GelMA profoundly suppressed NETs formation (Figure 6D). These comprehensive results demonstrate that mPDA‐PEI@GelMA not only inhibits the pro‐inflammatory polarization of neutrophils but also curtails NETs generation.

### 2.6 The mPDA‐PEI@GelMA Reduces Wound Inflammatory Response And Promotes Wound Healing in Streptozotocin‐Induced Diabetic Mice In Vivo

**Figure 7:**
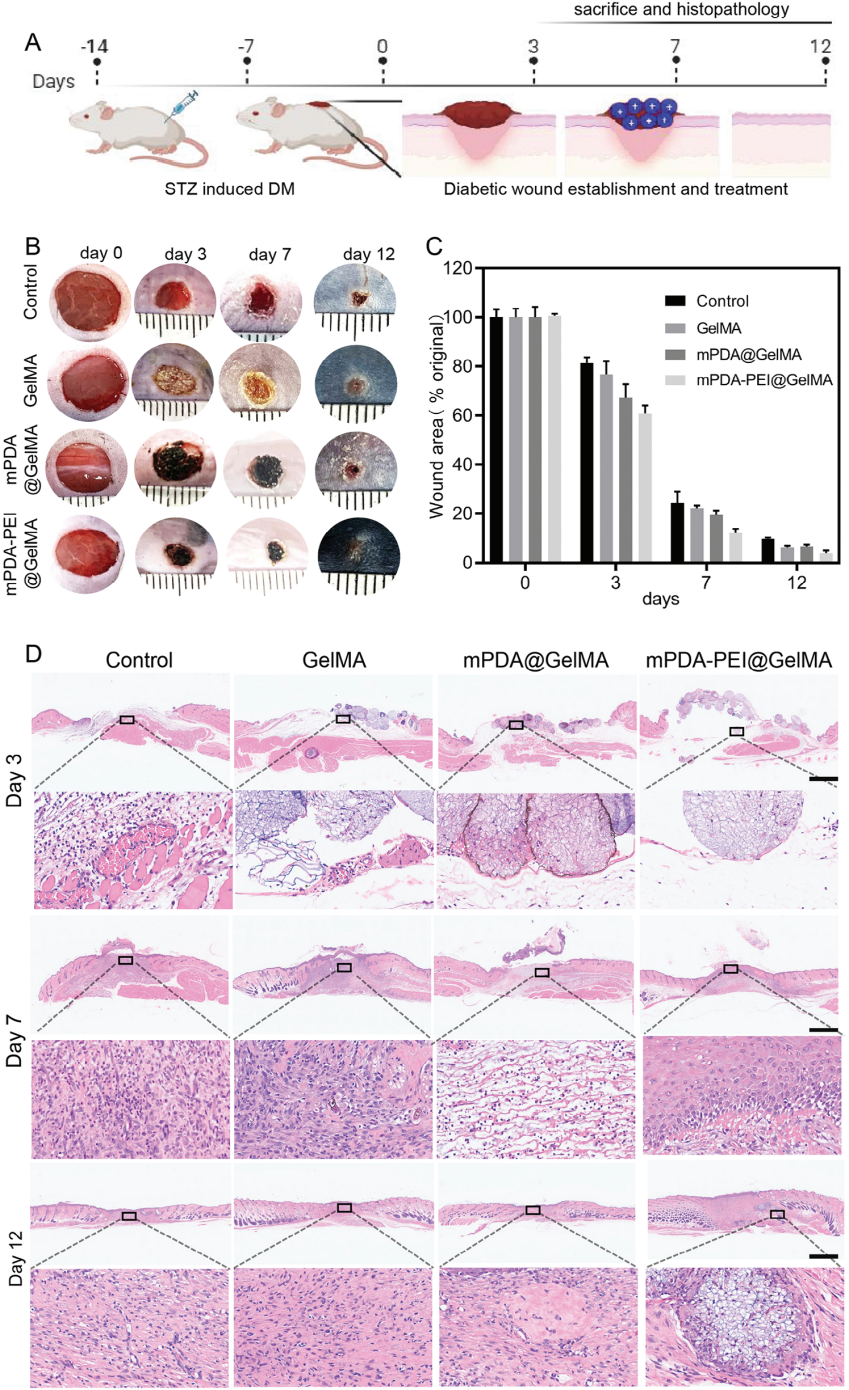
The effects of mPDA‐PEI@GelMA on diabetic wound healing in vivo. (A) Schematic illustration depicting the acceleration of diabetic wound healing based on mPDA‐PEI@GelMA scaffolds. (B) Representative photographs of wound healing with different treatment groups. (C) Wound healing rates in different treatment groups on days 0, 3, 7, and 12. (D) HE staining images of wound samples in different treatment groups at different time points. Data (mean ± SD) were quantified from three independent experiments, and statistical analysis was performed using one‐way ANOVA with a Tukey's posthoc multiple comparison test (*p< 0.05, **p< 0.01).

Building upon the findings that mPDA‐PEI@GelMA exhibits superior and ROS scavenging efficiency, thereby modulating inflammatory responses, neutrophil polarization, and NETs formation in vitro, we conducted an in vivo assessment of its efficacy in diabetic wounds healing using a diabetic full‐thickness skin defect model (**Figure** **7**A), using the diabetic mice models established by administering consecutive intraperitoneal injections of streptozotocin (STZ) until exhibiting a blood glucose level exceeding 16.7 mmol L^−1^ (Figure S12, Supporting Information). The progression of full‐thickness cutaneous wound healing at 0, 3, 7, and 12 days post‐surgery is depicted in Figure 7B. Throughout the healing process, no severe infection was observed, with varying healing rates and conditions noted across groups. Notably, wounds treated with mPDA‐PEI@GelMA exhibited a significantly accelerated healing process at each time point. By day 7, wounds in the control group remained moist with incomplete scabs and exudation, whereas wounds treated with GelMA, mPDA@GelMA, or mPDA‐PEI@GelMA displayed evident dryness and scab formation, likely due to the superior secretion absorption by the GelMA hydrogel microspheres, aiding in wound surface dryness. After 12 days, the mPDA‐PEI@GelMA group showed near‐complete wound closure, contrasting with the larger wound areas in the control, GelMA, and mPDA@GelMA groups (Figure 7B,C). This suggests the enhanced wound healing capabilities of mPDA‐PEI@GelMA, outperform the other groups.

Histological analyses, including HE staining, provided further insight into the biological effects on wound healing (Figure 7D). The mPDA‐PEI@GelMA group exhibited faster re‐epithelization and granulation tissue formation during remodeling, displaying more abundant and better‐conditioned granulation tissue compared to other groups. Additionally, the mPDA‐PEI@GelMA wounds displayed new granulation tissue and complex epidermal structures at day 12, including skin appendage‐like tissues. Notably, hydrogel microspheres integrated into the new skin tissue without eliciting significant inflammatory responses, confirming their safety and efficacy in wound repair.

**Figure 8:**
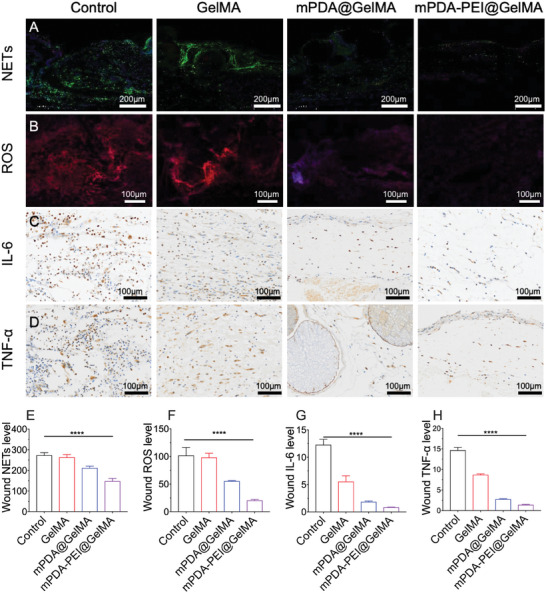
The effects of mPDA‐PEI@GelMA on NETs generation and inflammation modulation during diabetic wound healing in vivo. (A) Immunofluorescence staining of NETs in the mice wound from different groups on day 3. (B) Immunofluorescence staining of ROS from different groups. Levels of (C) IL‐6 and (D) TNF‐α in the wound from different groups on day 3. The relative mean fluorescence intensity of NETs (E) and ROS (F) was calculated by IntDen/Area using Image J software. The relative average optical density intensity of (G) IL‐6 and (H) TNF‐α was calculated by IntDen/Area using Image J software. Data (mean ± SD) were quantified from three independent experiments, and statistical analysis was performed using one‐way ANOVA with a Tukey's post hoc multiple comparison test (*p<0.05, **p<0.01, ***p<0.001, ****p<0.0001).

Chronic diabetic wounds often face impediments in healing due to persistent and disordered inflammation, characterized by excessive NETs formation‐a key obstacle in the healing process.^[^ ^31^ ^]^ Therefore, we further detected the effects of mPDA‐PEI@GelMA on excessive NETs formation in diabetic wounds. The control group exhibited over‐produced NETs, and application of mPDA‐PEI@GelMA significantly reduced NETs accumulation in diabetic wounds (**Figure** **8**A), a reduction more significant than that observed in the mPDA@GelMA and GelMA groups. Crucial to wound healing is the macrophage phenotypic transition from pro‐inflammatory (M1) to anti‐inflammatory (M2) types‐a process often disrupted in diabetic wounds.^[^ ^31^ ^]^ Immunofluorescence staining for inducible nitric oxide synthase(iNOS)/CD206 revealed a decrease in M1 macrophages and an increase in M2 macrophages in the mPDA‐PEI@GelMA group, indicating a shift toward an anti‐inflammatory state (Figure S13A,B, Supporting Information). Correspondingly, inflammatory cytokines IL‐6 and TNF‐α levels were significantly lower in the mPDA‐PEI@GelMA group (Figure 8C,D).

**Figure 9:**
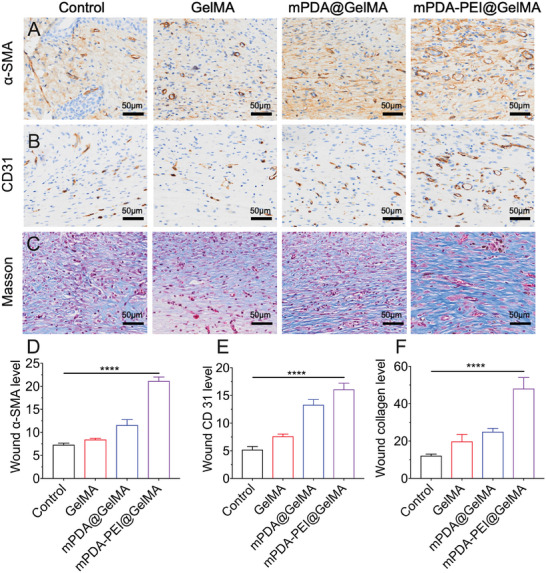
Immunofluorescence staining of (A) α‐SMA, (B) CD 31, and (C) Masson staining in the mice wound from different groups on day 12. Scale bars, 50 µm. The relative average optical density intensity of (D) α‐SMA, (E) CD 31, and (F) collagen deposition was calculated by IntDen/Area using Image J software. Data (mean ± SD) were quantified from three independent experiments, and statistical analysis was performed using one‐way ANOVA with a Tukey's post hoc multiple comparison test (*p<0.05, **p<0.01, ***p<0.001, ****p<0.0001).

Additionally, immunohistochemical staining for α‐smooth muscle actin (α‐SMA) and CD31 highlighted increased angiogenesis in the mPDA@GelMA and mPDA‐PEI@GelMA groups, particularly in the latter (**Figure** **9**A,B,D,E). Masson staining on day 12 revealed more collagen fiber regeneration in the mPDA‐PEI@GelMA group than in others (Figure 9C,F).

These comprehensive findings underscore the potential of mPDA‐PEI@GelMA in significantly reducing inflammation and enhancing diabetic wounds healing through its dual NETs and ROS scavenging abilities. Hence, bio‐based NETs scavengers like the ‘micro‐cage’ show promise for chronic wound healing and could represent an ideal therapeutic strategy for inflammation modulation.

## 3 Conclusion

This study has documented elevated NETs levels in wound exudation from patients with diabetic wounds, establishing a correlation with inflammatory indicators. Introducing a wound‐specialized hydrogel microspheres, termed a NETs scavenger ‘micro‐cage’ mPDA‐PEI@GelMA, this research aims to achieve non‐contact NETs scavenging between nanomaterials and the wound surface to enhance a wound healing. The mPDA‐PEI@GelMA effectively mitigates pro‐inflammatory responses associated with diabetic wounds by scavenging NETs and ROS. This action not only alleviates chronic inflammation but also accelerates wound healing in a diabetic mouse model. Additionally, mPDA‐PEI@GelMA demonstrates a regulatory effect on the neutrophil system within diabetic wounds, notably inhibiting the N1 phenotype of neutrophils and the generation of NETs. Consequently, mPDA‐PEI@GelMA emerges as a novel and promising treatment option for the management of diabetic wounds healing. The development of this NETs scavenger ‘micro‐cage’ opens avenues for innovative therapeutic approaches in addressing chronic inflammation and advancing the field of diabetic wounds care.

## 4 Experimental Methods

### Clinical Samples

Thirty‐three wound exudation samples were collected from 22 burn patients and 12 diabetic wounds patients, and 15 blood samples were donated by healthy volunteers in the Department of Burn Surgery, the Changhai Hospital of Naval Medical University. The collection of wound exudation and blood samples was approved by the ethics committee of Changhai Hospital (CHEC2021‐067). All patients and healthy donors were informed of the purpose of the donated wound exudation and blood samples and signed informed consent forms. Both the blood samples and wound exudation were centrifuged at 3000 rpm for 10 min at 4 °C, and then the supernatant was divided into sterile tubes and stored at −80 °C. The concentrations of cfDNA, cfmiRNA, IL‐6, MPO, TNF‐α, IL‐1β, and IFN‐γ in plasma and wound exudation were measured using the Quant‐iT PicoGreen dsDNA and miRNA Kits (Invitrogen, CA, USA) and corresponding ELISA kits (Thermo Fisher, Waltham, MA), respectively, following the manufacturer's protocols.

### Cit H3‐DNA ELISA

To quantify NETs in wound exudation, a capture ELISA based on histone H3 associated with DNA was used in this work.^[^ ^32^ ^]^ For the capture antibody, 1 µg mL^−1^ anti‐histone H3 (citrulline R2 + R8 + R17) antibody (ab5103, Abcam) was coated onto 96‐well plates (dilution 1:1000 in 100 µL) overnight at 4 °C. After washing three times, the plates were then blocked with 5% BSA for 2 h. 20 µL of samples and 80 µL incubation buffer containing a peroxidase‐labeled anti‐DNA mAb (Cell Death ELISAPLUS, Roche; dilution 1:25) was then added to the wells. After incubating for 2 h at room temperature, the plates were then washed by three times (300 µL each), and 100 µL peroxidase substrate (ABTS) was then added. After 20 min incubation at room temperature in the dark, 100 µL ABTS stop solution was then added and the plates were measured at 405‐nm wavelength.

### Animal Study

The animal experiments were conducted in accordance with the guidelines and regulations approved by the ethics committee of Shanghai Rat&Mouse Biotech Co., Ltd (No. IACUC‐20220920(11)). An 8‐week‐old male C57BL/6 mice model with diabetes was established through intraperitoneal injection of streptozotocin (STZ) at a dosage of 55 mg kg^−1^ for 6 consecutive days. The mice were considered diabetic models when exhibiting a blood glucose level exceeding 16.7 mmol L^−1^. Subsequently, the mice were anesthetized, and 6 mm diameter wounds with full‐thickness excisional skin were created on the dorsal skin.^[^ ^33^ ^]^ Following this procedure, the diabetic mice with wounds were randomly divided into four different treatment groups: the control group, GelMA group, mPDA@GelMA group, and mPDA‐PEI@GelMA group. The loading of nanoparticles (mPDA or mPDA‐PEI) by GelMA microspheres was 50 µg mg^−1^. At day 0,3,7,12 after surgery, the microspheres were replaced and evenly distributed across the entire wound. Digital photographs of wounds were taken regularly, and the wound healing rate was calculated using the following equation: wound healing rate (%)  =  (1–unhealed wound area/original area) × 100%.

### Histological Analysis and Immunohistochemistry

The wounds with healthy margins were excised on day 3, 7 and 12 after wounding and treatment, fixed in 10% formalin, and then embedded in paraffin for hematoxylin and eosin (H&E) staining and immunohistochemistry staining. The wound bed area of each section stained with H&E was measured using Image‐Pro Plus software. For evaluation of the number of M1 and M2 macrophages in day 3 wounds, immunofluorescence staining was performed with iNOS (Abcam) or CD206 (Abcam) to identify M1 or M2 macrophages, respectively. Immunofluorescence staining with Cit‐H3 (Abcam, ab5103, USA) and MPO (Proteintect, China) was carried out to detect NETs formation in the wounds of different treatment groups.

### DNA Binding Assay

cfDNA in wound exudation or water binding efficiency of materials (mPDA, GelMA, mPDA@GelMA or mPDA‐PEI@GelMA) was measured using Quant‐iT PicoGreenTM dsDNA Assay Kit (Invitrogen, CA, USA). In brief, the diluted Picogreen reagent and dsDNA in 1×TE buffer were initially mixed and incubated for 10 min in the dark at room temperature. Subsequently, different concentrations of materials materials (mPDA, GelMA, mPDA@GelMA, or mPDA‐PEI@GelMA) were added to the mixture. For cfDNA in wound exudation binding efficiency, the wound exudation and materials (mPDA, GelMA, mPDA@GelMA, or mPDA‐PEI@GelMA) were first incubated for 10 min, and then the diluted Picogreen reagent was mixed and incubated for 30 min in the dark at room temperature. The fluorescence intensity was measured using a Multiwall Plate Reader, and the binding efficiency of the materials to dsDNA was calculated.

### In Vitro TLR Activation Assays

HEK‐BlueTM hTLR3, hTLR8, and hTLR9 reporter cells were cultured in Dulbecco's modified eagle medium (DMEM) supplemented with 10% Foetal Bovine Serum (FBS) and 1% penicillin‐streptomycin following the instructions. To inhibit TLR3, 8, and 9 activation by materials (mPDA, GelMA, mPDA@GelMA, or mPDA‐PEI@GelMA), HEK‐Blue hTLRs reporter cells in DMEM were respectively plated at a density of 5 × 10^4^, 4 × 10^4^ and 8 × 10^4^ cells per well in a 96‐well plate for 30 min. Subsequently, 2 µL of Poly (I:C) (LMW), ORN 06, and CpG BW006 at 1 µg mL^−1^ or 10 µL wound exudation were added, followed by the addition of materials (mPDA, GelMA, mPDA@GelMA or mPDA‐PEI@GelMA) in a final volume of 200 µL. After incubating for 24 h, 50 µL of supernatants were collected and incubated with 150 µL of QUANTI‐Blue solution. The embryonic alkaline phosphatase (SEAP) activity in each well was measured by detecting the OD at 620 nm using a microplate reader.

### Cellular Localization of cfDNA and Materials

RAW264.7 of 2 × 10^4^ cells per well were initially cultured on confocal slides in a 24‐well plate. After 24 h, FITC‐labeled CpG (1 µg mL^−1^) and Cy5‐mPDA (100 µg mL^−1^), or Cy5‐mPDA‐PEI (100 µg mL^−1^) were added together. Following another 24 h of incubation, the supernatant was discarded, and cells were washed with PBS three times. Subsequently, a medium containing LysoTracker Red (Thermo Fish, Waltham, USA) was added and incubated for 15 min. Following the incubation, cells were washed and fixed using 4% paraformaldehyde at room temperature for 30 min. The nucleus was dyed with 1 × DAPI and the slide was sealed with neutral gum. Digital photographs were taken using a Confocal laser scanning microscopy to visualize the cellular localization of cfDNA and materials.

### Flow Cytometry

The inhibition of CpG DNA endocytosis by materials (mPDA@GelMA, mPDA‐PEI@GelMA, or GelMA) was measured using RAW264.7 cells. Briefly, after seeded RAW264.7 cells reached 70–80% confluence in a 24‐well plate, FITC‐labeled CpG DNA (1 µg mL^−1^) and materials (GelMA, mPDA@GelMA or mPDA‐PEI@GelMA, 2 mg mL^−1^) were added. After 24h of incubation, cells were harvested, and the fluorescence intensity of FITC‐CpG was measured using flow cytometry (MoFlo XDP, Beckman, USA). The neutrophil polarization to the N1 subtype induced by diabetic wounds exudation was measured using human peripheral blood primary neutrophils. This was done by detecting their cell surface marker, CD 95, CD 54, and CD11b, after co‐incubation with diabetic wounds exudation and materials (GelMA, mPDA@GelMA, or mPDA‐PEI@GelMA). Human neutrophils at a density of 2 × 10^5^ per well were co‐incubated with plasma obtained from healthy donors or wound exudation from diabetic wounds patients, with or without materials (GelMA, mPDA@GelMA or mPDA‐PEI@GelMA, 2 mg mL^−1^) for 4h at 37 °C. Subsequently, the cell surface markers CD 95, CD 54, and CD11b were detected using flow cytometry (MoFlo XDP, Beckman, USA).

### Ex vivo Human NETs Formation Assay

Ex vivo NETs formation was conducted using diabetic wound exudation. Human peripheral blood primary neutrophils obtained from Zhejiang Meisen Cell Technology Co., LTD, China, were used. Neutrophils at a density of 2 × 10^5^ per well were incubated with plasma obtained from healthy donors or diabetic wound exudation to induce NETs formation. To investigate the inhibition effect of NETs formation by materials (GelMA, mPDA@GelMA or mPDA‐PEI@GelMA of 2 mg mL^−1^), they were then added to the neutrophils together with diabetic wounds exudation. After 4h, specific immunofluorescence staining for elastase (Abcam, ab254178, USA) and MPO (Proteintect, China) was performed to assess NETs formation.

### Statistical Analysis

Data were expressed as the mean ± S.D. Statistical differences between groups were assessed using Student's t‐test for comparisons between two groups or one‐way analysis of variance (ANOVA) for comparisons involving more than two groups. GraphPad Prism 8 was employed for statistical analyses. In the figures, asterisks denote the following *p* values: *p < 0.05, **p < 0.01, ***p < 0.001 and ****p < 0.0001.

## Conflict of Interest

The authors declare no conflict of interest.

## Supporting information

Supporting Information

## Data Availability

The data that support the findings of this study are available from the corresponding author upon reasonable request.
