# Supplementary material for: Innovative Bio‐based Hydrogel Microspheres Micro‐Cage for Neutrophil Extracellular Traps Scavenging in Diabetic Wound Healing
Source: Adv Sci (Weinh). 2024 Apr 6;11(21):2401195. doi: 10.1002/advs.202401195 (PMC11151043; doi:10.1002/advs.202401195)
Supplement: Supplementary file 1 — Supporting Information [file ADVS-11-2401195-s001.pdf]

ADVANCED SCIENCE
Open Access

Supporting Information

for *Adv. Sci.*, DOI 10.1002/advs.202401195

Innovative Bio-based Hydrogel Microspheres Micro-Cage for Neutrophil Extracellular Traps Scavenging in Diabetic Wound Healing

Yongqiang Xiao, Tao Ding, He Fang, Jiawei Lin, Lili Chen, Duan Ma, Tianyu Zhang, Wenguo Cui* and Jing Ma**

Supporting Information

Innovative Bio-based Hydrogel Microspheres Micro-Cage for Neutrophil Extracellular Traps Scavenging in Diabetic Wound Healing

Yongqiang Xiao, Tao Ding, He Fang, Jiawei Lin, Lili Chen, Duan Ma, Tianyu Zhang, Wenguo Cui*, and Jing Ma**

Y. Xiao, J. Lin, L.Chen, T. Zhang, and J. Ma
ENT Institute, Department of Facial Plastic and Reconstructive Surgery, Eye & ENT Hospital, Fudan University, Shanghai, 200031, China.

T, Ding, and W. Cui
Department of Orthopaedics, Shanghai Key Laboratory for Prevention and Treatment of Bone and Joint Diseases, Shanghai Institute of Traumatology and Orthopaedics, Ruijin Hospital, Shanghai Jiao Tong University School of Medicine, 197 Ruijin 2nd Road, Shanghai, 200025, P. R. China.

D, Ma
Key Laboratory of Metabolism and Molecular Medicine, Ministry of Education, Department of Biochemistry and Molecular Biology, School of Basic Medical Sciences, Fudan University, Shanghai, China

H. Fang
Department of Burn Surgery, the First Affiliated Hospital, Naval Medical University, Shanghai, 200433, China;

Keywords: diabetic wound, neutrophil extracellular traps, hydrogel microspheres, inflammation modulation, mesoporous polydopamine

Table of contents

1. Experimental 3

2. The features of diabetic wounds..7

3. Synthesis of HPT and ssHPT 10

4. Characterizations of GelMA, mPDA@GelMA and mPDA-PEI@GelMA.....................12

5. Animal study 15

6. References 17

1. Experimental

Materials: Dopamine hydrochloride (DA·HCl), 1,3,5-Trimethylbenzene (TMB, 97%), pluronic F-127 (F-127), ammonia solution (NH3·H2O, 25%), methoxypolyethylene glycol amine (mPEG-NH2, MW=2000), methylene blue (MB), tris(hydroxymethyl)aminomethane (Tris, 99.9%), branched polyethylene imine (PEI, M.W. 10,000, 99%), 2-[4-(2-hydroxyethyl)-1-piperazinyl] ethanesulfonic acid (HEPES), fluorescein isothiocyanate (FITC, 90%), and H2O2 (30%) were all purchased from Aladdin (Shanghai, China). Cell counting kit-8 (CCK-8) assay, 4',6-diamidino-2-phenylindole (DAPI), phosphate buffered saline (PBS), dulbecco's modified eagle medium (DMEM) culture medium, antibiotic/antimycotic solution, fetal bovine serum (FBS), and 2,7-dichlorofluorescein diacetate (DCFH-DA) were purchased from Solarbio (Beijing, China). Murine ELISA Kit, ORN 06, CpG DNA, and Poly(I:C) were all purchased from Invitrogen (US). Neutrophil elastase antibody (ab254178) and MPO polyclonal antibody (22225-1-AP) were purchased from Abcam (UK) and Proteintech (China).

Methods.

Synthesis of mPDA NPs: mPDA was synthesized using a one-pot method based on a previously reported procedure.[1] In brief, F127 (0.36 g) and TMB (0.36 g) were initially dissolved in a mixture of H_2O (65 mL) and ethanol (60 mL). After stirring for 30min, 90mg TRIS was dissolved in 10ml H_2O solution and added to the mixture. Subsequently, 60mg of dopamine hydrochloride was added, and the reaction mixture was stirred at room temperature for 24 h. The resulting particles were separated by centrifugation and washed with ethanol and acetone. Template removal was performed by extraction, where the sample underwent ultrasonic treatment (3 times for 30 minutes each) in a mixture of ethanol and acetone (2:1 v/v). The final product was suspended in ethanol for further use.

Synthesis of mPDA-PEI NPs: The modification of mPDA with PEI was achieved through Michael addition/ Schiff base reactions. To modify mPDA with PEI, 1 mL PEI solution (1

mg/mL) was added to the particle suspension (1 mg/mL in 1 mL HEPES buffer, pH 7.4). The mixture was stirred at room temperature for 24 h in the absence of light. Subsequently, the reaction mixture was centrifuged and washed three times with water to obtain the nanoparticles.

Characterization of mPDA and mPDA-PEI: The morphological characteristics of mPDA and mPDA-PEI were assessed using Field Emission Transmission Electron Microscopy (FTEM, FEI, Holland). Zeta potentials were determined through a Zetasizer (Nano ZS90, Malvern Panalytical). Phase composition analysis of mPDA and mPDA-PEI was conducted using X-Ray Diffraction (XRD, PANalytical B.V., Holland) with a PANalytical X'Pert Powder diffractometer (Spectris Pte. Ltd, Netherlands), employing filtered Cu Kα radiation (λ= 1.5418 Å). Additionally, the chemical compositions of mPDA and mPDA-PEI were quantitatively analyzed via X-ray Photoelectron Spectroscopy (XPS) using an ESCALAB250Xi X-ray photoelectron spectrometer (Thermal Scientific, US).

Synthesis of GelMA: GelMA was prepared following a previously described method.[2] Briefly, 20g of gelatin was dissolved into 200 mL carbonate buffer solution (pH = 9) in a 50°C oil bath, resulting in a10% gelatin solution. Subsequently,2 mL methacrylic acid (MA) was added into gelatin solution (0.2 mL/min-1) using a syringe pump. After 3 h, 100 mL of PBS was added to halt the reaction. The resulting solution was then centrifuged and dialyzed at 38 °C for 48 h using a 3.5 kD dialysis bag. Following r lyophilization, pure GelMA products were obtained and stored at 4°C for subsequent experiments.

Fabrication of hydrogel microspheres: Porous GelMA microspheres were prepared using microfluidic technology, following procedures reported in previous studies.[3] The oil phase consisted of paraffin oil containing 10% Span 80 (v/v), while the aqueous phase is 5 wt% GelMA containing 0.5wt % photo-initiator. Both phases were injected using syringe pumps (Lead Fluid, China). The resulting GelMA microspheres were then frozen at −40°C and photo-crosslinked by ultraviolet (UV) light at 405 nm for 5 min. Then, the cross-linked

GelMA microspheres were centrifuged at 5000 rpm for 5 min and washed three times using ether and ddH_2O, respectively, to eliminate residual paraffin oil. The final step involved freeze-drying, resulting in white powder. This powder was sterilized under UV light and stored at 4°C for future use. For the preparation of mPDA-PEI @GelMA, the same method was employed, with the only difference being that the aqueous phase consisted of 5 wt% GelMA containing 0.5wt % photo-initiator and mPDA-PEI NPs (2 mg/mL).*Synthesis of mPDA-PEI @GelMA:* Following the preparation of GelMA microspheres, they were immersed in a simulated marine environment (10 mm Tris, 2mg/mL mPDA or mPDA-PEI, pH 8.5) and stirred for 12h. Subsequently, excess mPDA or mPDA-PEI was washed away. The formation of mPDA@GelMA or mPDA-PEI@GelMA was achieved by absorbing mPDA or mPDA-PEI onto the microspheres through immersion adsorption activity.

Physical Characterization of mPDA-PEI@GelMA: The particle size and general morphology of GelMA, mPDA@GelMA, and mPDA-PEI@GelMA were initially investigated and analyzed under bright field microscopy (Zeiss, Germany). To further assess the pore size and surface morphology, lyophilized GelMA, mPDA@GelMA, and mPDA-PEI@GelMA were observed by a scanning electron microscope (SEM, S-400, Japan). For a three-dimensional perspective, the morphology of Cy5-PEG labeled mPDA-PEI@GelMA was examined using onfocal laser scanning microscopy (Leica, DMI3000B, Germany).

•OH Radical Scavenging Assay: The •OH radical scavenging activities of materials (mPDA, mPDA-PEI, GelMA, mPDA@GelMA and mPDA-PEI@GelMA) were measured using the ABTS method described previously.[4] In brief, 920 μL of H_2O, varying concentrations of mPDA (0−100 μg/mL), mPDA-PEI NPs (0−100 μg/mL),, GelMA (2 mg/mL), mPDA@GelMA or mPDA-PEI@GelMA (2 mg/mL, equivalent concentration of mPDA or mPDA-PEI NPs was 100 μg/mL) , 10 μL of H_2O_2 (200 μM), and 20 μL of $FeSO_4 \cdot 7H_2O$ (18 mM) were mixed separately and were sonicated for 5 minutes. The supernatant was collected

after centrifugation and incubated with 50 μL ABTS (10 μM) for 3-5 min. Finally, the UV−vis absorbance at 800 nm was measured to evaluate the antioxidant activity of materials.

O2•− Radical Scavenging Assay: The O2•− radical scavenging activities of materials (mPDA, mPDA-PEI, GelMA, mPDA@GelMA and mPDA-PEI@GelMA) were measured by detecting the photoreduction inhibition ratio of nitroblue tetrazolium (NBT). Initially, 12.5 mM methionine, 75 μM NBT, and 20 μM riboflavin were mixed with varying concentrations of mPDA (0−100 μg/mL), mPDA-PEI NPs (0−100 μg/mL), GelMA (2 mg/mL), mPDA@GelMA or mPDA-PEI@GelMA (2 mg/mL, equivalent concentration of mPDA or mPDA-PEI NPs was 100 μg mL) in 1 mL of PBS (25 mM) solution. The mixture was then exposed to ultraviolet light for 1h. Finally, the UV−vis absorbance at 560 nm was calculated to evaluate the O2•− radical scavenging activities of materials.

DPPH Radical Scavenging Activity: Varying concentrations of mPDA (0-100 μg/mL), mPDA-PEI (0-100 μg/mL), GelMA (2 mg/mL), mPDA@GelMA or mPDA-PEI@GelM (2 mg/mL, equivalent concentration of mPDA or mPDA-PEI NPs was 100 μg/mL) was suspended into 2,2-diphenyl-1-picryl-hydrazyl-hydrate (DPPH), respectively and incubated for 20 min. After centrifugation, the absorbance at 517 nm was measured.

2. The features of diabetic wounds

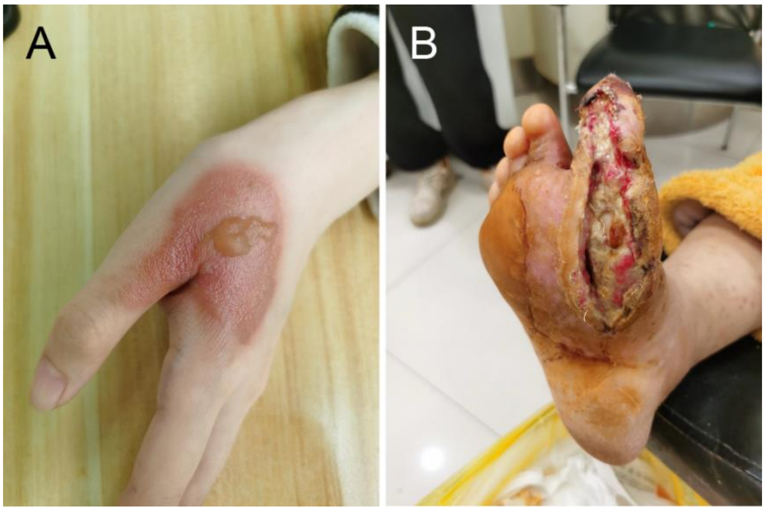

Figure S1. The images of patients with (A) burn wounds or (B) diabetic chronic wounds.

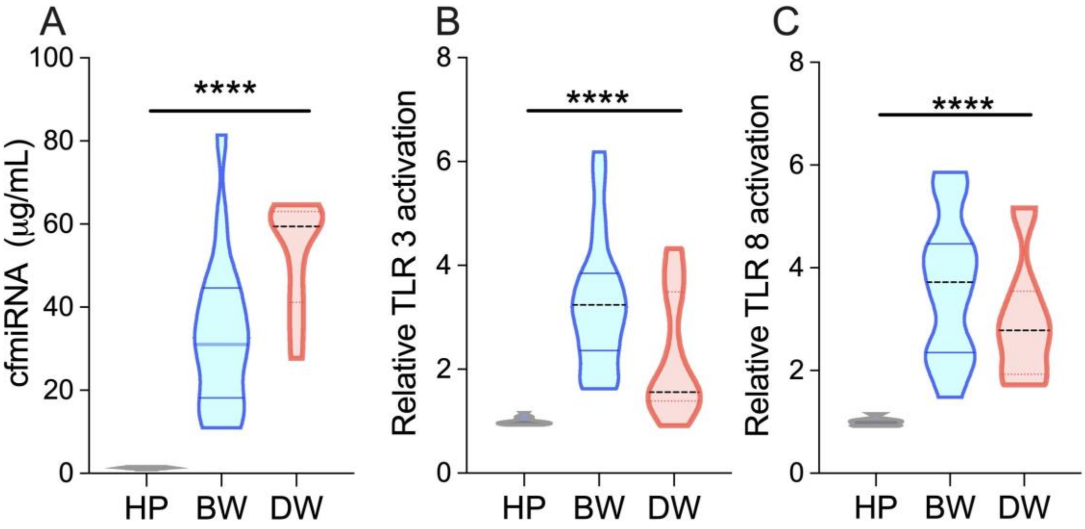

Figure S2. (A) The cfmiRNA concentration in plasma from healthy volunteers (n=15) and wound exudation from patients with burn wounds (n=21) and diabetic wounds (n=12). Data represent the mean ± S.D. (One-way ANOVA, *p<0.05, **p<0.01, ***p<0.001, ****p<0.001). Activation of (B) TLR 3 and (C) TLR 8 reporter cells by plasma from healthy volunteers (n=15) and wound exudation from patients with burn (n=21) and diabetic (n=12). Data represents the mean ± S.D. (One-way ANOVA, *p<0.05, **p<0.01, ***p<0.001, ****p<0.0001).

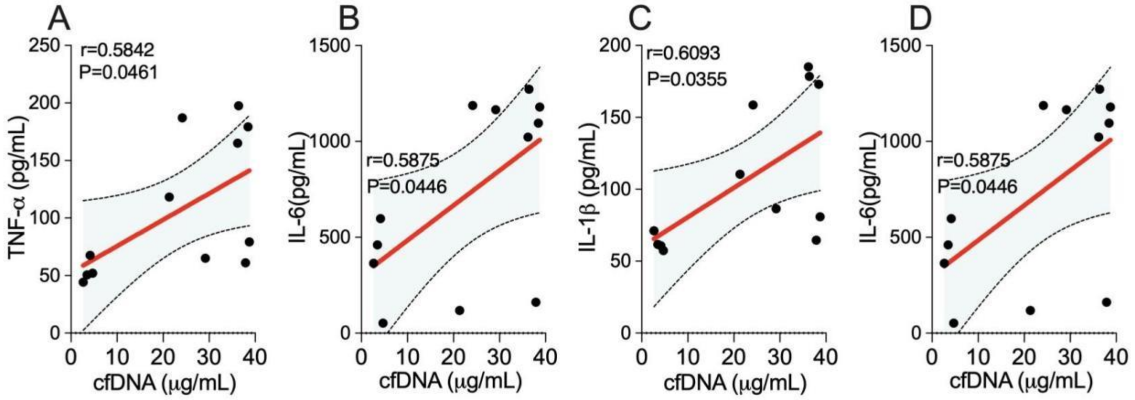

Figure S3. The correlation between cfDNA concentration and TNF-α (A), IL-6 (B), IL-1β (C) and IFN-γ (D) levels in wound exudation s of patients with diabetic wounds. The red line is the fitted regression line, and the light blue shading around it is the 95% confidence interval. Coefficient ‘r’ represents Spearman’s correlation between cfDNA levels and TNF-α, IL-6, IL-1β and IFN-γ in the wound exudation s of patients with diabetes (n=12) .

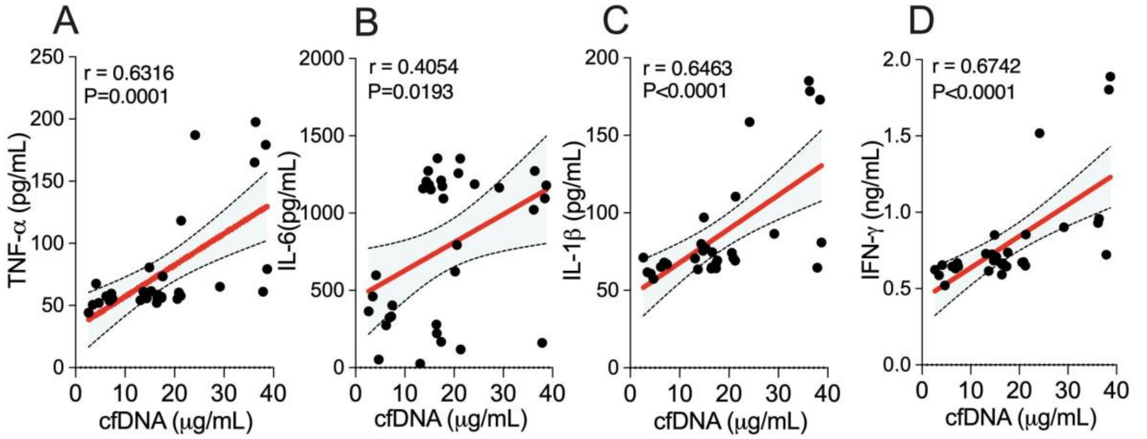

Figure S4. The correlation between cfDNA concentration and TNF-α (A), IL-6 (B), IL-1β (C) and IFN-γ (D) levels in wound exudation s of patients with burn and diabetic wounds. The red line is the fitted regression line, and the light blue shading around it is the 95% confidence interval. Coefficient ‘r’ represents Spearman’s correlation between cfDNA levels and TNF-α, IL-6, IL-1β and IFN-γ (n=33) .

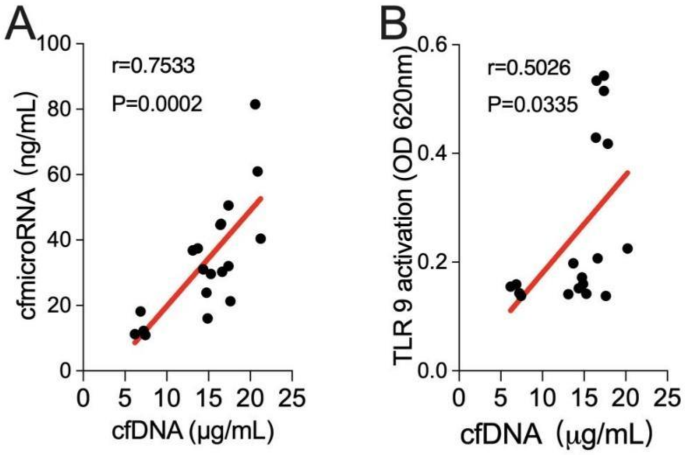

Figure S5. The correlation between cfDNA concentration and cfmicroRNA (A) level and TLR 9 (B) activation level by HEK-TLR 9 cells in the wound exudation of patients with diabetic (n=12).

3. The cfDNA binding efficiency of mPDA-PEI

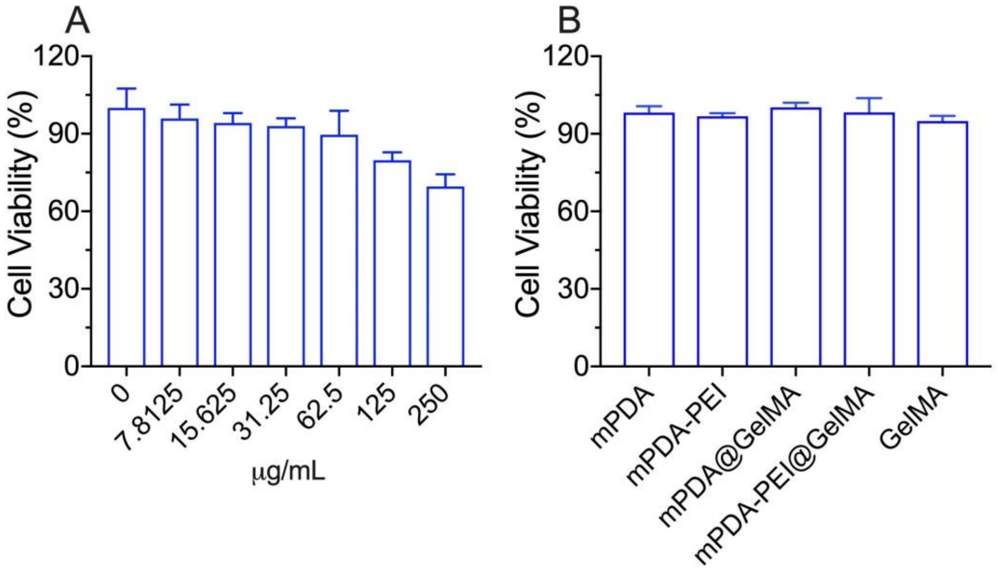

Figure S6. Cell viability of HDF after 24 h treatment with mPDA-PEI at different concentrations (A) and mPDA, mPDA-PEI, GelMA, mPDA@GelMA, and mPDA-PEI@GelMA (B).

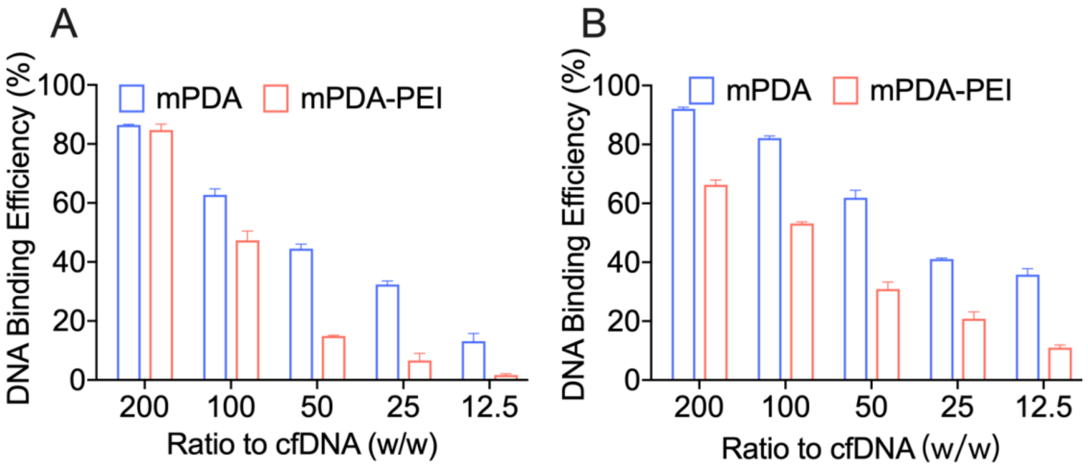

Figure S7. The cfDNA binding efficiency of mPDA and mPDA-PEI in water(A) and in 10% FBS(B).

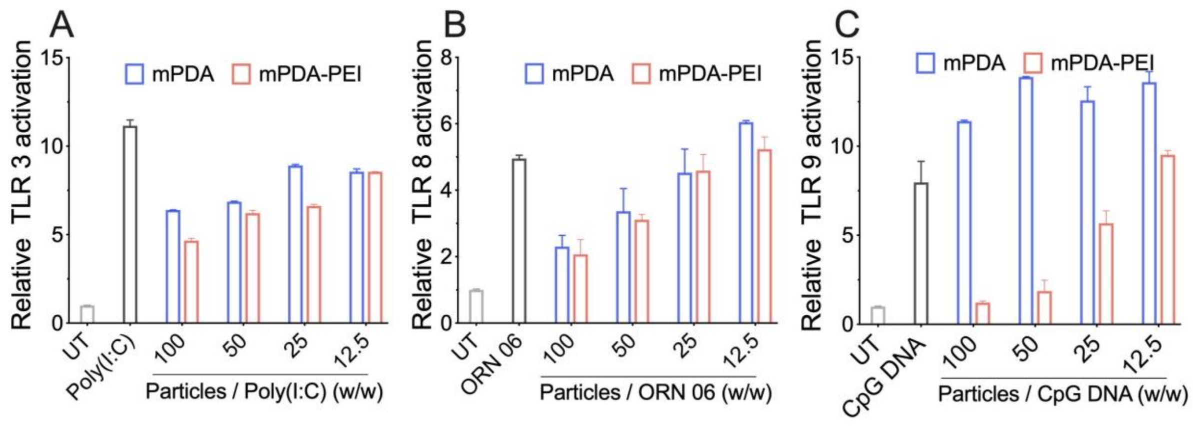

Figure S8. The inhibition efficiency of the activation of HEK-TLR 3, 8, and 9 reporter cells by Poly(I:C), ORN 06, or CpG DNA (1 μg/mL) of mPDA and mPDA-PEI.

4. Characterizations of GelMA, mPDA@GelMA and mPDA-PEI@GelMA

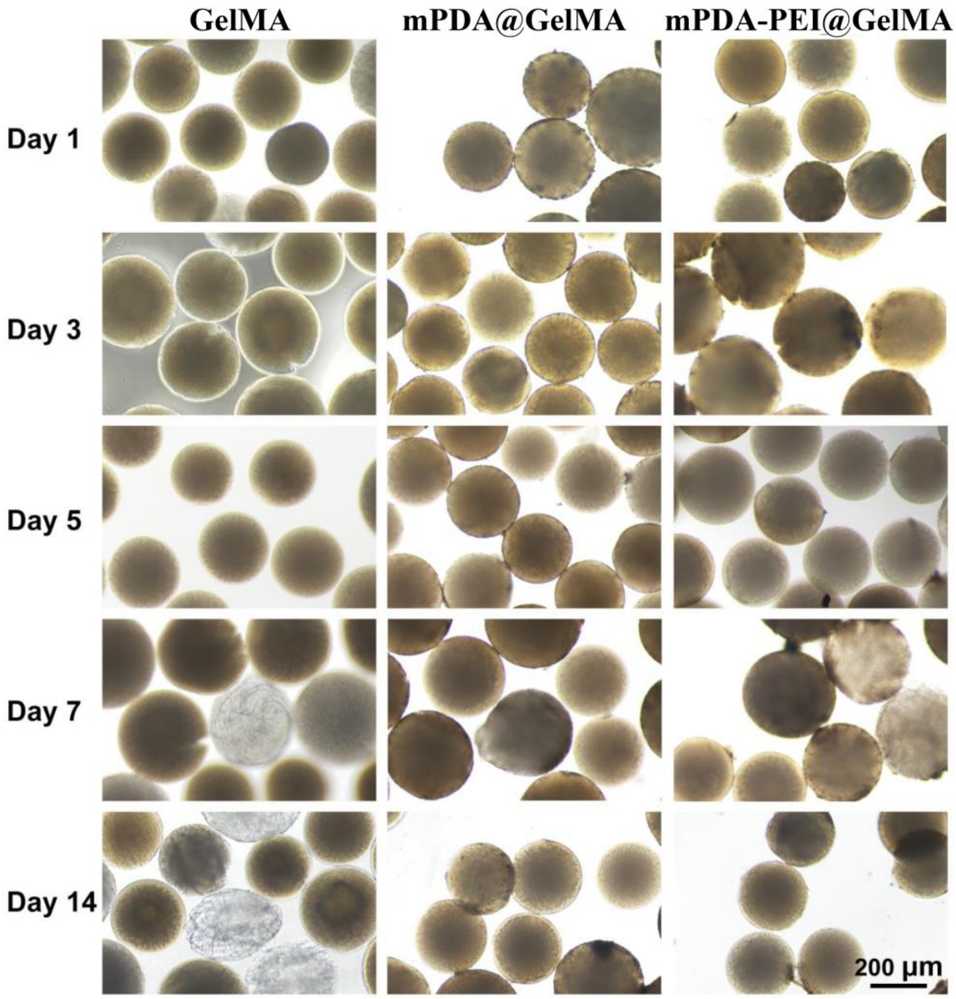

Figure S9. Images of GelMA, mPDA@GelMA, and mPDA@GelMA within 2 weeks at 37°C in PBS (pH=7.4).

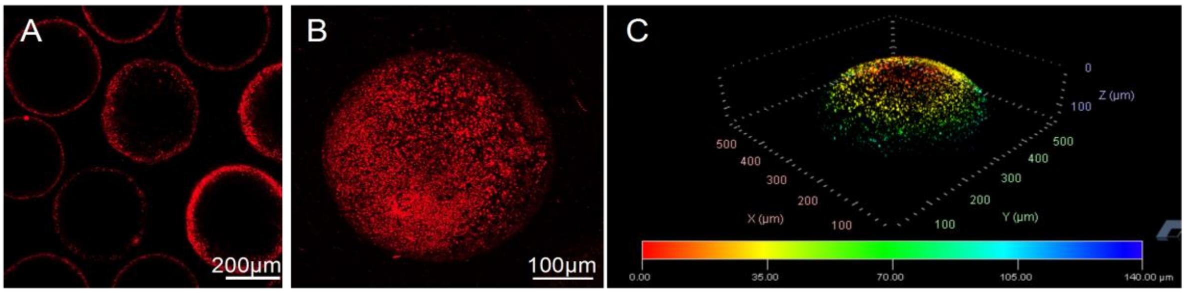

Figure S10. Fluorescent images of GelMA hydrogel microspheres engineered Cy5.5-mPDA nanoparticle.

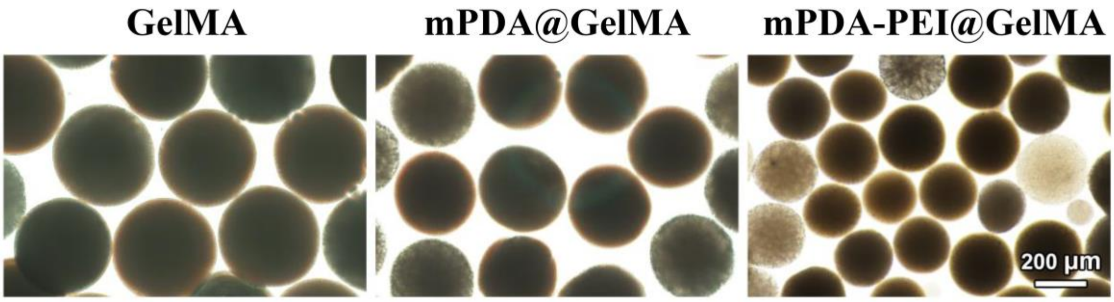

Figure S11. The general morphology of GelMA, mPDA@GelMA, and mPDA-PEI@GelMA.

5. Animal study

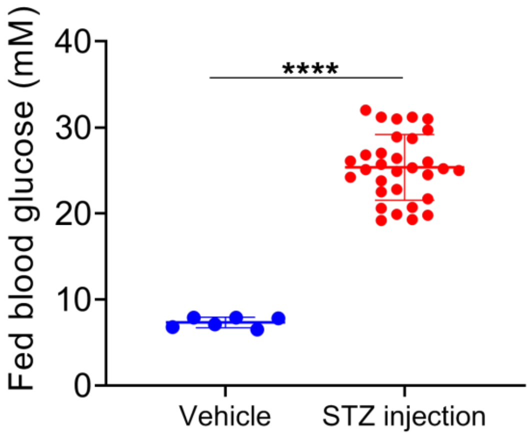

Figure S12. The fed blood glucose of male C57BL/6 mice one week after intraperitoneal injection of streptozotocin (STZ) at a dosage of 55 mg/kg for 6 days continuously.

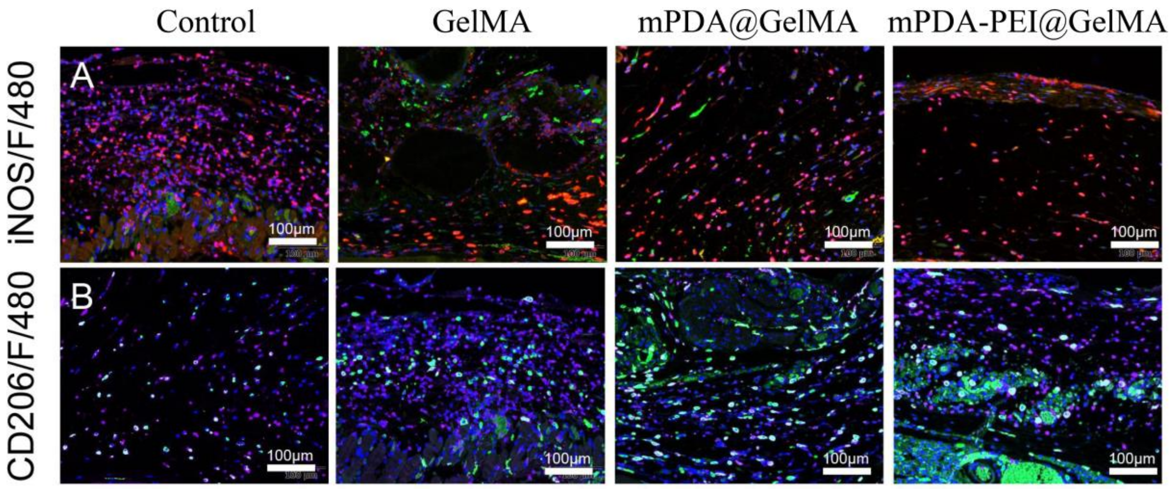

Figure S13. (A) M1 and (B) M2 macrophage levels in the wound from different groups on day 3.

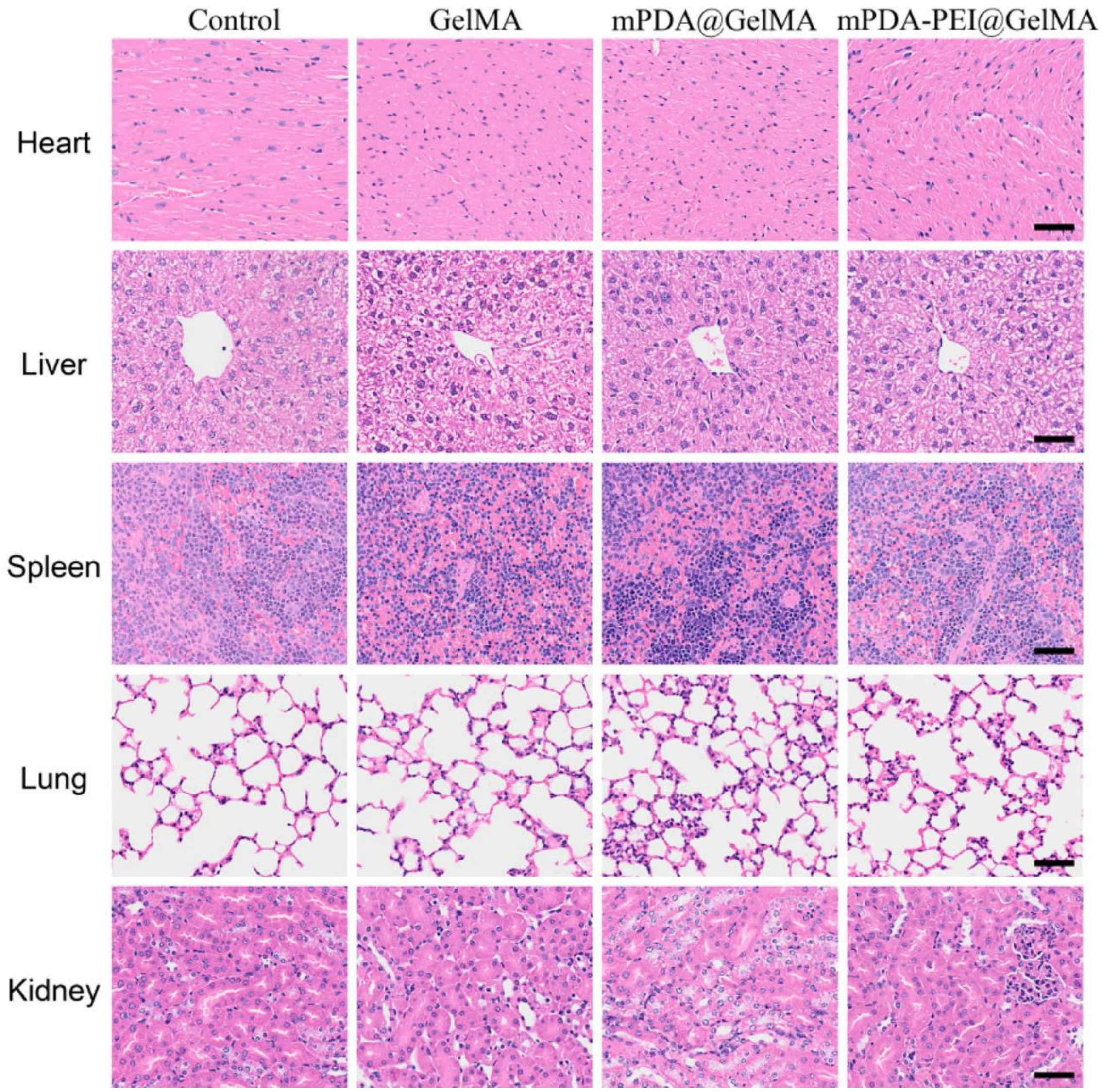

Figure S14. H&E staining of hearts, livers, spleens, lungs, and kidneys from mice after different treatments. Scale bars, 50 μm.

6. References

[1] F. Chen, Y. Xing, Z. Wang, X. Zheng, J. Zhang, K. Cai, *LANGMUIR* **2016**, *32* (46), 12119, https://doi.org/10.1021/acs.langmuir.6b03294.

[2] H. Shirahama, B. H. Lee, L. P. Tan, N. J. Cho, *Sci Rep* **2016**, *6*, 31036, https://doi.org/10.1038/srep31036.

[3] K. Chen, F. Wang, R. Ding, Z. Cai, T. Zou, A. Zhang, D. Guo, B. Ye, W. Cui, M. Xiang, *Small* **2022**, *18* (36), e2106591, https://doi.org/10.1002/smll.202106591.

[4] A. Elkamhawy, N. K. Oh, N. A. Gouda, M. H. Abdellattif, S. O. Alshammari, M. A. S. Abourehab, Q. A. Alshammari, A. Belal, M. Kim, A. A. Al-Karmalawy, K. Lee, *Metabolites* **2023**, *13* (2), https://doi.org/10.3390/metabo13020141.
